# COVID-19-Induced Postural Orthostatic Tachycardia Syndrome and Dysautonomia

**DOI:** 10.7759/cureus.40235

**Published:** 2023-06-10

**Authors:** Resnah Minhas, Adithya Sateesh Bharadwaj

**Affiliations:** 1 Medicine, American University of Antigua, St. Johns, ATG; 2 Medicine, University of Maryland Midtown Campus, Baltimore, USA

**Keywords:** chest pain, auto-immune neuropathy, generalized anxiety disorder, tachycardia, autoimmune pots

## Abstract

Postural orthostatic tachycardia syndrome (POTS) is a disorder characterized by orthostatic intolerance and, by definition, includes clinical symptoms of lightheadedness, palpitations, and tremulousness among others. It is considered a relatively rare condition that affects approximately 0.2% of the general population, and it is estimated that between 500,000 to 1,000,000 individuals in the United States have the condition and recently has been linked to post-infectious (viral) etiologies. We present a case of a 53-year-old woman who was diagnosed with POTS following extensive autoimmune workup, who was also status post-severe acute respiratory syndrome coronavirus 2 (SARS-CoV-2) infection. The post-coronavirus disease 2019 (COVID-19) cardiovascular autonomic dysfunction can affect global circulatory control, which describes increased heart rate even at resting states, and local circulatory disorders, such as coronary microvascular disease leading to vasospasm, as described by the patient’s chest pain, and venous retention leading to pooling and reduced venous return after standing. Along with tachycardia with orthostatic intolerance, other symptoms can also accompany the syndrome. In the majority of patients, intravascular volume is reduced, leading to decreased venous return to the heart and causing reflex tachycardia and orthostatic intolerance. Management varies from lifestyle modifications to pharmacologic therapy, to which patients generally show a good response. POTS should be a differential on the cards, especially in patients post-COVID-19 infection, as these symptoms can be misdiagnosed as having psychological etiologies.

## Introduction

Postural orthostatic tachycardia syndrome (POTS) is a disorder characterized by orthostatic intolerance and, by definition, includes clinical symptoms of lightheadedness, palpitations, tremulousness, generalized weakness, blurred vision, exercise intolerance, and fatigue [1]. The condition is characterized by an elevated heart rate (>30 beats per minute) upon transitioning from a supine or sitting position to a standing position, despite the absence of orthostatic hypotension [1]. It is often confused and misdiagnosed as anxiety, panic disorder, or chronic fatigue syndrome due to the similarity of symptoms shared among these diseases [1]. POTS is considered a relatively rare condition that affects approximately 0.2% of the general population, and it is estimated that between 500,000 to 1,000,000 individuals in the United States have the condition. It is more common in women than men, and it typically develops in adolescence or early adulthood (ages 15-25 at diagnosis), although the disorder can affect individuals of any age or sex [1]. Recently, several case reports showed an increasing incidence of POTS as a complication of severe acute respiratory syndrome coronavirus 2 (SARS-CoV-2) infection in patients who had chronic signs and symptoms of the infection (long COVID (coronavirus disease)). Patients with the chronic sequelae of the coronavirus infection presented with a multitude of symptoms, including shortness of breath, palpitations, fatigue, cognitive impairment (brain fog), sleep disturbance, orthostatic intolerance, peripheral neuropathy symptoms, chest and abdominal discomfort, nausea, diarrhea, joint and muscle pains, anxiety or depression symptoms, skin rashes, sore throat, headache, earache, and tinnitus [2]. A clinical presentation of orthostatic tachycardia along with some or all of the aforementioned signs and symptoms of long COVID can indicate a diagnosis of POTS as a complication of COVID-19 infection [2].

## Case presentation

**Figure 1:**
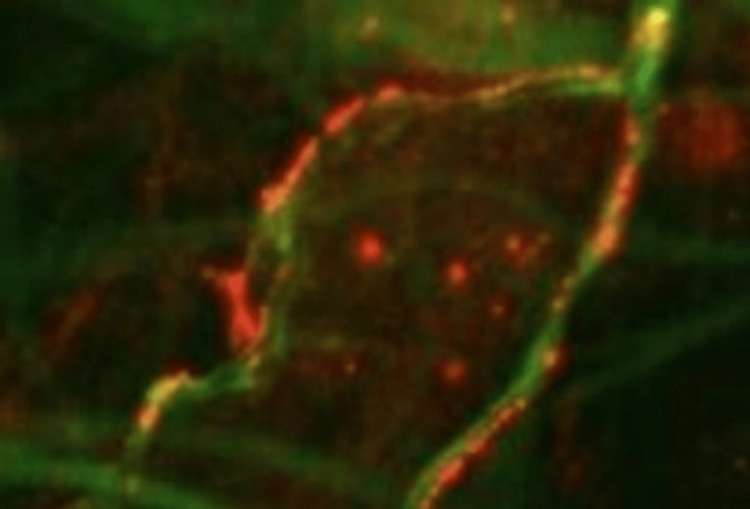
Biopsy showing regions of phosphorylated alpha-synuclein on cutaneous nerves

A 53-year-old female with a past medical history of asthma, osteoarthritis, and status post-COVID-19 infection presented to her cardiologist due to symptoms of shortness of breath, chest pain, fatigue, tachycardia, and brain fog. The patient had been experiencing shortness of breath and chest pain since the COVID-19 infection and was previously diagnosed with COVID-induced pneumonia. The patient stated that her heart rate would spike to 120 beats per minute after walking across the room according to her smartwatch. This, according to the patient, was abnormal for her since prior to COVID, she was very active and exercised daily. However, she was limited due to extreme fatigue and sharp, stabbing chest pain after initiating exercise. She also mentioned feeling warm and flushed on standing up, and symptoms improved when sitting down during these episodes. She also had a single episode of syncope and mentioned symptoms of neuropathic pain in her upper extremities. The patient denied orthopnea, paroxysmal nocturnal dyspnea, lower extremity edema, palpitations, claudication, presyncope, and syncope. Vitals and physical exam were within normal limits. A cardiac stress echocardiogram performed at the time revealed no abnormalities and a normal ejection fraction of 63%. Simultaneously, she had also been following up with pulmonology and was on a trial of albuterol 108 MCG/ACT inhaler and fluticasone propionate 50 MCG/ACT nasal spray, which did not improve her symptoms. The patient’s lab values, including pulmonary function tests (PFT), cardiac event monitoring, thyroid stimulating hormone (TSH), brain natriuretic peptide (BNP), computerized tomography of the chest, and erythrocyte sedimentation rate (ESR), were normal. Her rheumatoid factor was elevated, confirming a possibility of an autoimmune component to her syndrome. A small fiber neuropathy skin biopsy evaluation indicated positive pathologic evidence of phosphorylated alpha-synuclein deposition within cutaneous nerves (Figure 1). A detailed summary is provided in Table 1. The postganglionic sympathetic cholinergic sudomotor function test revealed decreased sweat volume at the proximal leg. Stress echocardiography was normal. Electrocardiogram (EKG) taken during each visit was normal. She was also placed on cardiac monitoring, which was uneventful. The exaggerated cardiac response to activity, especially with a change in posture led to a working diagnosis of postural orthostatic tachycardia syndrome.

**Table 1:** Description of phosphorylated alpha-synuclein deposition on each biopsy site

Biopsy site	Phosphorylated alpha-synuclein deposition	Description
Posterior Cervical (Right)	Abnormal	Two or more colocalized fibers seen across all stained sections
Distal Thigh (Right)	Abnormal	One colocalized fiber seen across all stained sections
Distal Leg (Right)	Normal	No phosphorylated alpha-synuclein depositions observed in stained sections

The patient was asked to follow up monthly and to start a structured exercise program. Over periods of follow-up visits to her cardiologist, she still complained of a heart rate of up to 120 beats per minute after standing up to walk. Subsequently, she was suggested to try a trial of medical therapy, including ivabradine 5 mg 2x daily. She reported a decrease in fatigue and tachycardia symptoms, however, she still mentioned chest discomfort and flushing of the skin. Her symptoms fluctuated between her tri-monthly doctor’s visits. Later in the course of treatment, she was given lisdexamfetamine 30 mg by her neurologist to aid in memory and brain fog. Fludrocortisone 0.1 mg and a high sodium diet pre-exercise were added to her management to help with retaining adequate blood volume. Ivadabrine was eventually discontinued after the patient felt her heart rate was too low, according to her watch, and thought the fatigue was worse than when she started. She was also referred to an allergy clinic for possible workup of mast cell activation or autoimmune subtypes of POTS. The impression of the allergy clinic was post-COVID dysautonomia with POTS and possibly small fiber neuropathy. This patient’s diagnosis of POTS was made by her cardiology clinic after a referral from her primary care physician. POTS was diagnosed clinically with features presenting for more than six months with an orthostatic heart rate increase of more than 30 beats/minute within 10 minutes of standing and the absence of significant orthostatic hypotension. On follow-up, the patient is relatively clear of POTS symptoms with occasional flare-ups of tachycardia. She claimed ivabradine helped her overall, with progressive upright exercises. The patient still has chest pain, which her cardiologist believes may be an endothelial lining issue.

## Discussion

**Figure 2:**
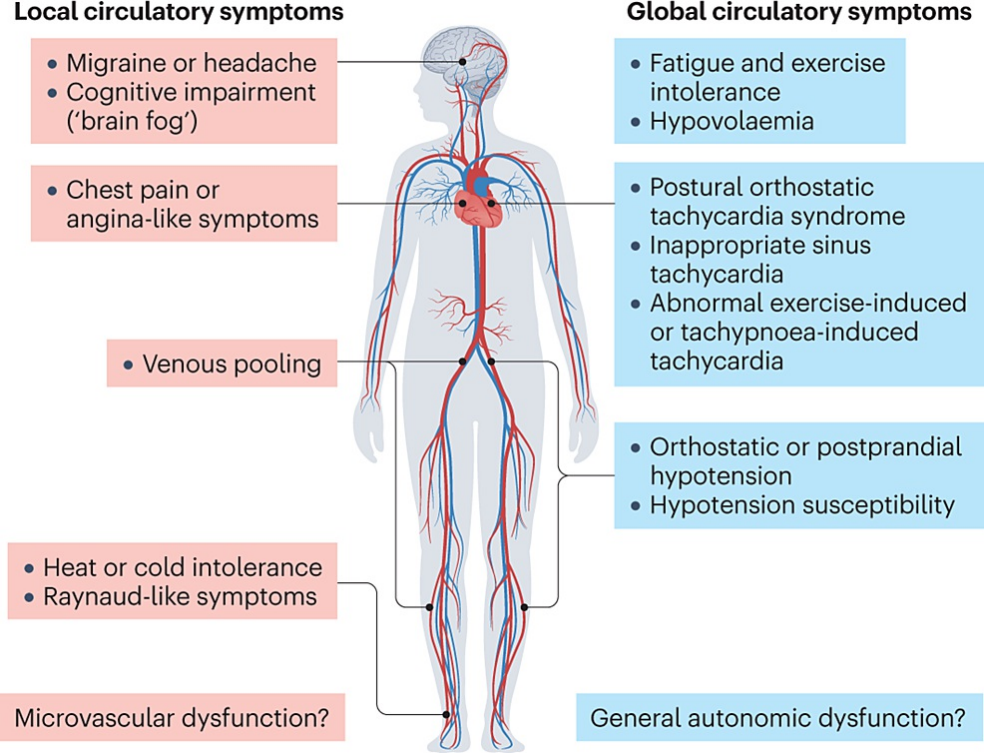
Explaining common multiorgan manifestations of postural orthostatic tachycardia syndrome

POTS and other cardiovascular dysautonomias can develop after a SARS-CoV-2 infection. It is thought that these factors might be potent immune triggers that can evoke an autoimmune response in individuals who are susceptible. Additionally, the severity of the infection is reported to be unrelated to the occurrence of these symptoms [3]. The post-COVID-19 cardiovascular autonomic dysfunction can affect global circulatory control, which describes increased heart rate even at resting states, and local circulatory disorders, such as coronary microvascular disease leading to vasospasm, as described by the patient’s chest pain, and venous retention leading to pooling and reduced venous return after standing (Figure 2) [4]. Along with tachycardia with orthostatic intolerance, other symptoms can also accompany the syndrome. In the majority of patients, intravascular volume is reduced, thus leading to decreased venous return to the heart and causing reflex tachycardia and orthostatic intolerance. Patients who have been on prolonged bed rest or are in a deconditioned state are likely to develop decreased blood volume [5]. For instance, one study showed healthy volunteers confined to bed rest for two weeks developed orthostatic intolerance along with a 17% reduction in plasma volume and a 5% reduction in cardiac mass [6]. Many patients may avoid physical activity due to the exacerbation of orthostatic intolerance, however, reconditioning efforts often improve but do not fully restore function or eliminate symptoms in POTS [7].

Some patients have shown evidence of increased adrenergic function separate from tachycardia, suggesting that POTS represents a hyperadrenergic state. Commonly, patients with POTS have increased catecholamine levels than healthy controls [8]. Studies have found that patients with POTS have, on average, higher levels of plasma angiotensin II and lower levels of plasma renin and aldosterone [9]. Correspondingly, higher levels of angiotensin II may reflect a deficiency in the vasodilatory effects of endothelial nitric oxide also found in some POTS patients [10]. A small fiber neuropathy may be present in up to 50% of POTS cases and contributes to dysautonomia as seen in our patient. An autoimmune basis for a small number of POTS patients has been linked to the observation of orthostatic symptoms following a viral illness [11]. Additionally, autoantibodies to the ganglionic nicotinic acetylcholine receptor and alpha 1 adrenergic, beta 1 and 2 adrenergic, and angiotensin II type 1 receptors have been detected in a few patients. These autoantibodies are speculated to impair effective peripheral vasoconstriction leading to compensatory tachycardia and further research is warranted to understand their exact mechanisms [12]. Multiple case series have reported POTS symptoms as a delayed manifestation of COVID-19 infection in some patients who had no prior history of symptoms [13].

Autonomic testing is useful in the diagnosis of POTS in patients with orthostatic intolerance symptoms and for a reassessment of changing symptoms over time. Autonomic testing is more sensitive and specific than random measurement of heart rate during clinical bedside assessment while standing [14]. Examples of this include using vasomotor adrenergic autonomic testing such as a tilt table test. This provides objective evidence of orthostatic intolerance in patients with symptoms of lightheadedness. It increases the diagnostic yield of evaluating tachycardia in patients with POTS and helps exclude other possible etiologies such as orthostatic hypotension. Sudomotor testing is also used, as a majority of POTS patients have neuropathic abnormalities. This test demonstrates the effectiveness of postganglionic sudomotor nerves. A study was conducted to determine the relationship of sudomotor abnormalities to other aspects of dysautonomia in POTS. Fifty-six percent (56%) of patients had an abnormal QSART (quantitative sudomotor axon reflex testing), as in this patient. which was typically patchy and involved the lower extremity. No differences in autonomic tests or spectral indices were observed between hyperadrenergic and nonhyperadrenergic POTS [14]. Laboratory tests should be used to exclude other causes of symptoms or additional findings. Important test values to consider are morning cortisol, transthoracic echocardiography, and 24-hour urinary sodium excretion if hypovolemia is suspected (values less than 100 mEq/24 hours suggest hypovolemia) [15]. Tryptase should also be evaluated with prominent flushing to screen for a mast cell activation disorder.

The management of POTS relies on symptom management through intravascular volume expansion, physical exercise, and lifestyle changes as well as compressive garments. Oral fluid intake should be aimed at 3L daily with a daily salt intake of about 8-12 g of sodium chloride to help reduce hypovolemia [16]. Incremental aerobic exercise programs should be implemented for all patients with POTS. Despite the patient’s intolerance to upright position exercises, patients have shown progress in a recumbent or semi-recumbent position such as recumbent biking, swimming, or machine rowing. The exercise program should include lower extremity resistance strength training to help reduce venous pooling. A small study involving patients with POTS who were assigned to a three-month exercise training program (consisting of multiple days a week for about an hour) showed improved cardiovascular responses to exercise, lower heart rates, and faster heart rate recovery after exercise compared to baseline levels [17]. Supervised training may be required for those who cannot adhere to an exercise program independently. The aim of these sessions is to reach about 70-75% of the maximal predicted heart rate. As fitness improves, patients should be able to gradually increase the frequency, duration, and intensity of exercise and advance to exercising in an upright position [18]. Lifestyle modifications can be utilized to provide adjunctive benefits to many patients. Patients should be encouraged to be upright during most of the day and avoid prolonged bed rest. A physical habit that can be implicated is improving venous return by contracting the skeletal muscles in the leg. Examples include leg crossing, weight shifting, and lower extremity muscle tensing. Along with this, adjunctive treatment options include compressive garments and medication. Compressive garments, such as compressive stockings or abdominal binds, help reduce venous pooling. Abdominal compression may be more beneficial in reducing venous pooling because of the higher venous capacitance provided by the splanchnic mesenteric bed. It is also more adhered to by patients than compressive stockings because it can be applied when the patient is upright and is readily removed at other times. This helps improve compliance with the compressive garment [19]. Initial medication is started to target the underlying symptoms that may exacerbate or worsen a patient's current condition. Beta-blockers such as metoprolol and propranolol are beta-adrenergic antagonists that are used to blunt elevations in the heart rate and thus may be used for patients with POTS. Midodrine is an alpha-adrenoreceptor agonist that is used to constrict arteriolar and venous capacitance vessels and reduces blood pooling. However, it must be used with caution as it can cause supine hypertension. In a non-blinded trial of adolescents with POTS, midodrine at 2.5 mg once daily was proven to be superior to metoprolol 0.5 mg/kg daily or increased salt and water intake, with 89% efficacy during three to six months of follow-up despite the very low dose used [20,21]. Ivadabrine is a selective “funny” (I f) current inhibitor, thus lowering the heart rate. Several case series have been reported lowering the heart rate and symptomatic improvement in more than half of patients treated with ivadabrine at 2.5 mg to 20 mg/day in one to two doses. In a small crossover trial of patients with POTS, those who were given Ivadabrine at 2.5 mg to 7.5 mg twice daily, had a reduced heart rate, and improved symptoms compared to those assigned to the placebo [22]. The overall prognosis of POTS is not directly clear. Although it is a chronic condition and may appear throughout life; it does not increase the risk of mortality but does affect the patient’s activities of daily life. Many state that POTS improves spontaneously with or without treatment. There is no evidence to currently support one form of treatment over another. Lifestyle modifications and pharmacotherapy are the choices available and patients respond to each treatment modality differently. Sometimes, relief is brought via a combination of these therapies.

## Conclusions

Postural orthostatic tachycardia syndrome (POTS) is a disorder characterized by orthostatic intolerance and, by definition, includes clinical symptoms of lightheadedness, palpitations, and tremulousness, among others. Recently, POTS has been associated with patients who were infected with the SARS COV-2 virus and usually affects these patients after the recovery period. The presentation of symptoms can vary on a case-by-case basis; however, POTS should be a differential on the cards, especially in patients post-COVID-19 infections, as these symptoms can be misdiagnosed as other conditions such as generalized anxiety or chronic fatigue syndrome. Management varies from lifestyle modifications to pharmacologic therapy to which patients generally show a good response.
